# Safety Evaluation of a Novel Strain of *Bacteroides fragilis*

**DOI:** 10.3389/fmicb.2017.00435

**Published:** 2017-03-17

**Authors:** Ye Wang, Huimin Deng, Zhengchao Li, Yafang Tan, Yanping Han, Xiaoyi Wang, Zongmin Du, Yangyang Liu, Ruifu Yang, Yang Bai, Yujing Bi, Fachao Zhi

**Affiliations:** ^1^Institute of Genetic Engineering, Jinan University,Guangzhou, China; ^2^Guangdong Provincial Key Laboratory of Gastroenterology, Department of Gastroenterology, Institute of Gastroenterology, Nanfang Hospital, Southern Medical University,Guangzhou, China; ^3^State Key Laboratory of Pathogen and Biosecurity, Beijing Institute of Microbiology and Epidemiology,Beijing, China; ^4^Guangzhou ZhiYi biotechnology Co. Ltd.,Guangzhou, China

**Keywords:** *Bacteroides fragilis*, safety evaluation, probiotic, whole genome sequencing, genetic stability

## Abstract

Commensal non-toxigenic *Bacteroides fragilis* confers powerful health benefits to the host, and has recently been identified as a promising probiotic candidate. We previously isolated *B. fragilis* strain ZY-312 and identified it as a novel strain based on 16S rRNA sequencing and morphological analyses. We also determined that ZY-312 displayed desirable probiotic properties, including tolerance to simulated digestive fluid, adherence, and *in vitro* safety. In this study, we aim to investigate whether ZY-312 meets the safety criteria required for probiotic bacteria through comprehensive and systematic evaluation. Consequently, the fatty acid profile, metabolite production, and biochemical activity of strain ZY-312 were found to closely resemble descriptions of *B. fragilis* in Bergey’s manual. Taxonomic identification of strain ZY-312 based on whole genome sequencing indicated that ZY-312 and ATCC 25285 showed 99.99% similarity. The 33 putative virulence-associated factors identified in ZY-312 mainly encoded structural proteins and proteins with physiological activity, while the lack of *bft* indicated that ZY-312 was non-toxigenic. *In vivo* safety was proven in both normal and immune-deficient mice. The 11 identified antibiotic resistance genes were located on the chromosome rather than on a plasmid, ruling out the risk of plasmid-mediated transfer of antibiotic resistance. *In vitro*, ZY-312 showed resistance to cefepime, kanamycin, and streptomycin. Finally, and notably, ZY-312 exhibited high genetic stability after 100 passages *in vitro*. This study supplements the foundation work on the safety evaluation of ZY-312, and contributes to the development of the first probiotic representative from the dominant Bacteroidetes phylum.

## Introduction

Commensal gut microbiota are important for host health. They contribute to maturation of the immune system, building intestinal commensalism, resisting infectious microbes, digesting indigestible carbohydrates, and producing certain nutrients such as vitamins and short-chain fatty acids (Sekirov et al., 2010; Fijan, 2014). An increasing number of reports have confirmed the link between intestinal flora disorder and disease, especially autoimmune and metabolic diseases (Fijan, 2014; Althani et al., 2016). Although it is not clear who takes the leap and how it happens, dominant members of the intestinal microbiota are now being examined as potential probiotic candidates to assist in the treatment of disease, or become alternatives to antibiotics (McKenney and Pamer, 2015; Miquel et al., 2015).

Approved probiotic products based on intestinal microbiota species mainly include lactic acid bacteria, *Bifidobacterium*, and *Escherichia coli* (Foligne et al., 2013). However, there are currently no probiotic representatives of the phylum Bacteroidetes, the second-largest component of the intestinal flora. Recently, non-toxigenic *Bacteroides fragilis* (NTBF) was shown to have powerful health benefits to the host, and was recommended as a probiotic candidate (Troy and Kasper, 2010; Hsiao et al., 2013; Deng et al., 2016). It is therefore likely that *B. fragilis* will be the first probiotic species from the phylum Bacteroidetes.

We previously isolated a novel *B. fragilis* strain ZY-312, and have carried out basic safety assessments to test its probiotic properties (Deng et al., 2016). Results of 16S rRNA sequence analysis, tolerance to simulated digestive fluid, safety, and adhesion to HT-29 cells suggested *B. fragilis* ZY-312 possessed desirable probiotic properties. However, these results are not sufficient to definitively conclude that ZY-312 is safe for use as a probiotic. According to the framework of evaluations for probiotics from Europe (Miquel et al., 2015) and by the Food and Agriculture Organization of the United Nations and the World Health Organization (FAO/WHO, 2002), thorough characterization of a strain, including examination of the fatty acid profile, metabolite production, and biochemical activity, evaluation of the potential risk of transferable antibiotic resistance and genetic stability, and verification of safety in animals, especially immunodeficient animals, is an integral part of safety assessment (Miquel et al., 2015). Taking advantage of whole genome sequencing technology, we carried out a thorough characterization and systematic evaluation of novel *B. fragilis* strain ZY-312 to determine whether it meets the safety criteria required for probiotics.

## Materials and Methods

### Bacterial Strains and Culture Conditions

Strain ZY-312 was isolated from the feces of a healthy infant, and was previously identified using 16S rRNA gene sequence analysis (Deng et al., 2016). *B. fragilis* strain ATCC 25285 (also known as NCTC 9343) was purchased from the American Type Culture Collection (ATCC). The culture conditions and materials were as described previously for strains ZY-312 and ATCC 25285 (Deng et al., 2016). For the microbial contamination experiment, *E. coli* CBSLAM00087, *Staphylococcus aureus* (*S. aureus*) CMCC(B) 26058, *Salmonella enterica (S. enterica)* serotype Paratyphi B CBSLAM00994, *Pseudomonas aeruginosa* (*P. aeruginosa*) CBSLAM00818, *Candida albicans (C. albicans)* CMCC(F)98001, and *Clostridium sporogenes* (*C. sporogenes)* CMCC(B) 64941 were obtained from the Academy of Military Medical Science, Beijing, China. All strains were verified by PCR amplification of 16S rRNA gene using the universal primers 27 F (5′-AGAGTTTGATCCTGGCTCAG-3′) and 1492 R (5′-GGTTACCTTGTTACGACTT-3′) (Lanes D–J). Amplified products were sequenced (Biomed).

### Animals

For acute oral toxicity studies, 6- to 8-week-old female specific pathogen-free (SPF) BALB/c mice and nude mice were procured from the Animal Experiment Center of the Academy of Military Medical Sciences, Beijing, China. The animals had free access to tap water and standard rodent diet. All of the animal experiments were performed in accordance with the approval of the Animal Ethics Committee of the Beijing Institute of Microbiology and Epidemiology, Beijing, China.

### Genome Sequencing, Assembly, and Analysis

Total DNA was extracted from all strains using a DNA extraction kit (Qiagen, USA) and the complete genome sequence of strain ZY-312 was determined using an Illumina Hiseq 2000 sequencing system (Xu et al., 2015). A genome sequencing library with an average insert size of 400 bp was generated, and raw short-read sequences were filtered using Seqprep^1^ and Sickle^2^ software. The genome was then assembled *de novo* using SOAP *de novo* software (Luo et al., 2012). Accuracy of the assembled genome sequence was evaluated by mapping all raw reads onto the scaffolds using SOAPaligner (Cui et al., 2013).

Gene prediction was carried out using Glimmer3.0 software (Xu et al., 2015). Putative antibiotic resistance genes and putative virulence factors were identified by BLAST analysis of the antibiotic resistance genes database (ARDB)^3^ (Liu and Pop, 2009) and the virulence factors database (VFDB)^4^ (Chen et al., 2005), respectively. A BLAST comparison was performed between the genome sequences of strain ZY-312 and *B. fragilis* strain NCTC 9343 (GenBank accession number NC_003228). A Perl script was written and the average nucleotide identity (ANI) was calculated using BLAST. A neighbor-joining phylogenetic tree was built using treebest based on the ZY-312 complete genome sequence, the complete shotgun sequences of other *B. fragilis* strains, and the complete genome sequences of *B. fragilis* strains NCTC 9343 (GenBank accession number NC_003228), YCH46 (GenBank accession number NC_006347), and *B. fragilis* 638R (GenBank accession number NC_016776).

### General Characteristics of ZY-312

ZY-312 was cultured on Eosin methylene blue agar for 24 h at 37°C (*E. coli* as positive control), SS agar for 24 h at 37°C (*S. enterica* serovar Paratyphi B as positive control), Columbia agar containing blood and gentamicin for 48 h at 37°C (*C. sporogenes* as positive control), mannitol sodium chloride agar for 24 h at 37°C (*S. aureus* as positive control), NAC agar for 24 h at 37°C (*P. aeruginosa* as positive control), Sabouraud dextrose agar for 72 h at 28°C (*C. albicans* as positive control), rose bengal agar for 96 h at 28°C (*C. albicans* as positive control), and agar agar for 48 h at 37°C (*S. aureus* as positive control) for excluding microbial contamination. The major fatty acids were analyzed using an HP6890 gas chromatograph (ver. A 5.01), as previously described (Tan et al., 2010). Supernatant from late-logarithmic phase ZY-312 and ATCC 25285 cultures was collected by centrifugation for 10 min at 3000 ×*g*, and then analyzed by gas chromatography-mass spectrometry. Carbon-source utilization analyses were carried out using a Biolog AN microPlate test panel (Biolog, USA). Bacterial cells were collected from logarithmic phase ZY-312 and ATCC 25285 cultures and resuspended at a concentration of 1.5 × 10^8^ colony forming units (cfu)/mL, and then inoculated into the test plate. After incubating anaerobically for 16–24 h, test results were read using a microplate reader (SpectraMax, M2), using deionized water as a negative control. To test catalase activity, 3–5 drops (about 100 μL) of 3% hydrogen peroxide (freshly prepared) were dropped onto the center of precipitated bacterial cells on microscope slides. To test gelatin liquefaction activity, bacteria were inoculated into gelatin medium, tryptone soy broth (TSB, OXIOD, UK) solidified with 15% gelatin, using a sterilized needle. Plates were incubated at 37°C for 48 h anaerobically, then placed at 4°C for 3–4 h. To determine hemolytic ability, bacteria were anaerobically cultured on tryptone soy agar (TSA, OXIOD, UK) supplemented with 5% (v/v) goat blood for 48 h at 37°C. To determine motility, bacteria were cultured on semi-solid medium (TSB solidified with 0.5% agar) at 37°C for 48 h anaerobically.

### Antibiotic Resistance Testing

Antibiotic resistance testing was performed using the minimum inhibitory concentration (MIC) method as described previously (Fernandez et al., 2005). Test antibiotics were chosen based on the antibiotic resistance genes identified in the annotated genome of ZY-312, with the following antibiotics included in the analysis: cefoxitin, ceftriaxone, cefepime, trimethoprim, clarithromycin, chloromycetin, levofloxacin, streptomycin, kanamycin, tetracycline, vancomycin, and polymyxin B (NIFDC, China). Although *bcrA* was identified in the genome, bacitracin was not used because of high toxicity, while fosmidomycin (*rosA*) is still undergoing testing and was therefore also excluded. Quinupristin/dalfopristin, the targets of the *vatB* gene product are not used in China, and there is no corresponding antibiotic for *ykkC*. ZY-312 was cultivated anaerobically at 37°C for 48 h at a concentration of 10^7^ cfu/mL with antibiotics at different concentrations. MIC was determined by measuring optical density at 600 nm (OD_600_) with a microplate reader (SpectraMax, M2).

### Acute Toxicity to Normal Mice and Nude Mice

To assess the acute toxicity of ZY-312, SPF BALB/c mice were randomly assigned into five groups (*n* = 5–8). Each group was administered with ZY-312 in a suspension containing 1 × 10^9^, 5 × 10^10^, or 5 × 10^11^ cfu/day, 0.5 mL/day culture supernatant, or 0.5 mL/day saline via oral gavage for 5 days. General condition and body weight were observed daily for 17–18 days. At the end of the experimental period, blood samples were obtained for hematological and serum biochemistry analyses. Stomach, colon, liver, and spleen were collected, weighed, and prepared for histopathological examination. Nude mice were orally administered with ZY-312 suspension at a dosage of 1 × 10^9^ cfu/day for 3 days. General condition and bodyweight were observed for 7 days.

### Genetic Stability

To explore whether genetic variation occurs in ZY-312 during *in vitro* passage, we continuously subcultured ZY-312 for 100 generations. Two parallel tubes were inoculated from an original culture of ZY-312 in TSB supplemented with 5% fetal bovine serum, and then incubated at 37°C in an anaerobic glove box (Bugbox, Ruskin) for 24 h. These initial cultures were designated A_0_ and B_0_. Subsequent passages were performed by inoculating 1% of each culture into fresh medium every 24 h, until A_100_ and B_100_ were obtained. Genetic variation was evaluated by complete genome sequencing of strains A_10_, A_25_, A_50_, and A_100_, and B_10_, B_25_, B_50_, and B_100_, and ANI was calculated for each generation. Morphological variations were observed by colony examination, Gram staining, and scanning electron microscopy (SEM) of A_100_ and B_100_, with wild-type ZY-312 used for comparison. Growth of ZY-312, A_100_, and B_100_ under anaerobic conditions was also examined over a 24-h period. Acute toxicity of A_100_ and B_100_ was detected in SPF mice at a dosage of 1 × 10^9^ cfu/day for 3 days, with general condition and bodyweight observed for 7 days.

### Experimental Replicates and Statistical Methods

All experiments were performed at least in triplicate using independent assays, and values were expressed as the mean ± standard error. An unpaired Student’s *t*-test was performed to determine statistically significant differences in the acute toxicity assays. A *p*-value of <0.05 was considered statistically significant.

## Results

### Morphological Characteristics

Strain ZY-312 only grew under anaerobic conditions (**Supplementary Figure S1**), with no growth observed under aerobic conditions or in 5% CO_2_, implying it was an obligate anaerobe. Microbial contamination was excluded by culturing ZY-312 in different culture media under different conditions (**Supplementary Figure S2**). ZY-312 formed circular, low convex, semi-opaque colonies following anaerobic cultivation on blood agar plates (**Supplementary Figure S1**). Cells were Gram-negative, and shown to be rod shaped with rounded ends by scanning electrochemical microscopy (**Supplementary Figure S1**). This morphology matched the descriptions of *B. fragilis* in Bergey’s manual (Krieg et al., 2001).

### General Characteristics

The major fatty acids of ZY-312 were C_15:0_ anteiso, C_15:0_ iso, C_16:0_, and C_16:0_ 3-OH (**Supplementary Figure S3** and **Table 1A**). The major products in the culture supernatant of ZY-312 were lactic acid, acetic acid, succinic acid, propionic acid, and phenylacetic acid (**Table 1B**). ZY-312 and ATCC 25285 differed in their ability to metabolize L-valine, salicin, L-alanine, L-alanyl-L-glutamine, and *N*-acetyl-D-glucosamine (**Table 2A**). Both strains were positive for catalase activity, weakly positive for gelatin liquefaction, and negative for hemolytic activity and motility (**Table 2B**). The physiological and biochemical characteristics of ZY-312 were in accordance with *B. fragilis* as described in Bergey’s manual (Krieg et al., 2001).

**Table 1:** Major fatty acids (A) and major metabolites (B) of *Bacteroides fragilis* ZY-312.

Peak name			Percentage (%)	
**(A)**				
C_15:0_ anteiso			32.21	
C_15:0_ iso			17.29	
C_16:0_			11.66	
C_16:0_ 3-OH			11.08	
C_14:0_			1.82	
C_17:0_ ante 3-OH			1.72	
C_18:0_			1.68	
C_15:0_			1.63	
C_18:1_ CIS 9			1.37	
Unable to distinguish			19.55	

	**ZY-312**		**ATCC 25285**	
	**Time (s)**	**Normalized area**	**Time (s)**	**Normalized area**

**(B)**				
Acetic acid	1514	67.24 × 10^5^	1513	27.65 × 10^5^
Butanedioic acid	593	498.29 × 10^5^	592	107.27 × 10^5^
propionic acid	1353	2.65 × 10^5^	1352	5.41 × 10^5^
Phenylacetic acid	2404	1.33 × 10^5^	2403	2.06 × 10^5^
lactic acid	424	556.78 × 10^5^	424	557.94 × 10^5^

**Table 2:** Physiological and biochemical properties of *B. fragilis* ZY-312.

	ZY-312	ATCC		ZY-312	ATCC
**(A)**					
Water	−	−	Dulcitol	−	−
*N*-Acetyl-D-galactosamine	+	+	D, L-α-Glycerol phosphate	−	−
*N*-Acetyl-D-glucosamine	/	+	*N*-Acetyl-β-D-mannosamine	−	−
D-Fructose	+	+	L-Fucose	−	−
Adonitol	−	−	D-Galactose	+	+
Amygdalin	+	+	D-Galacturonic acid	−	−
D-Arabitol	−	−	Gentiobiose	+	+
Arbutin	+	+	D-Gluconic acid	−	−
D-Cellobiose	+	+	D-Glucosaminic acid	−	−
α-Cyclodextrin	+	+	α-D-Glucose	+	+
β-Cyclodextrin	+	+	Glucose-1-phosphate	+	+
Dextrin	+	+	Glucose-6-phosphate	+	+
Glycerol	−	−	α-Methyl-D-galactoside	−	−
i-Erythritol	−	−	β-Methyl-D-galactoside	+	+
m-Inositol	−	−	α-Methyl-D-glucoside	−	−
α-D-Lactose	+	+	β-Methyl-D-glucoside	−	−
Lactulose	+	+	Palatinose	+	+
Maltose	+	+	D-Raffinose	+	+
Maltotriose	+	+	L-Rhamnose	−	−
D-Mannitol	−	−	Salicin	+	−
D-Mannose	+	+	D-Sorbitol	−	−
D-Melezitose	−	−	Stachyose	+	+
D-Melibiose	+	+	Sucrose	+	+
3-Methyl-D-glucose	+	+	D-Trehalose	−	−
Pyruvic acid methyl ester	+	+	D-Lactic acid methyl ester	−	−
Acetic acid	−	−	D-Malic acid	−	−
Formic acid	−	−	L-Malic acid	−	−
Fumaric acid	−	−	Propionic acid	−	−
Glyoxylic acid	−	−	Pyruvic acid	+	+
α-Hydroxybutyric acid	/	/	Turanose	+	+
β-Hydroxybutyric acid	−	−	D-Saccharic acid	−	−
Itaconic acid	−	−	Succinamic acid	−	−
α-Ketobutyric acid	+	+	Succinic acid	−	−
α-Ketovaleric acid	+	+	L-Asparagine	−	−
D,L-Lactic Acid	−	−	m-Tartaric acid	−	−
L-Lactic acid	−	−	Urocanic acid	−	−
L-Alaninamide	−	−	L-Methionine	−	−
L-Alanine	/	+	L-Phenylalanine	−	−
L-Alanyl-L-glutamine	+	/	L-Serine	−	−
L-Alanyl-L-histidine	/	/	L-Threonine	−	−
L-Alanyl-L-threonine	+	+	L-Valine	/	+
Succinic acid monomethyl ester	−	−	L-Valine plus L-aspartic acid	−	−
L-Glutamic acid	−	−	2′-Deoxy adenosine	+	+
L-Glutamine	−	−	Inosine	+	+
Glycyl-L-aspartic acid	+	+	Thymidine	+	+
Glycyl-L-glutamine	−	−	Uridine	+	+
Glycyl-L-methionine	−	−	Glycyl-L-proline	−	−
Thymidine-5′-monophosphate	−	−	Uridine-5′-monophosphate	−	/

	**ZY-312**	**ATCC 25285**			

**(B)**					
Catalase assay	Positive	Positive			
Gelatin liquefaction test	Weakly positive	Weakly positive			
Hemolysis test	Negative	Negative			
Dynamic test	Negative	Negative			

### Genetic Characteristics

The phylogenetic trees generated from the whole genome sequence of ZY-312 and other *B. fragilis* strains are shown in **Supplementary Figures S5**, **S6**. ZY-312 and ATCC 25285 had an ANI of 99.99%. In total, 33 putative virulence factors (**Table 3**) and 11 antibiotic resistance genes (**Table 4A**) were annotated in the ZY-312 genome based on a minimum of 40% amino acid homology. The complete genome was 4,558,494 bp in length and contained on a single chromosome, with an average GC content of 43.08%, consistent with that of *B. fragilis* (41–44%) (Krieg et al., 2001). All drug-resistance genes were located on the chromosome rather than on plasmids.

**Table 3:** Putative virulence-associated genes identified in the genome of *B. fragilis* ZY-312.

Gene ID	VFDB_ID	Name	Function
YDGL001582	VF0091	Alginate algI	Alginate O-acetyltransferase AlgI
YDGL004163	VF0003	Capsule cap8E	Capsular polysaccharide synthesis enzyme Cap8E
YDGL004164	VF0003	Capsule cap8G	Capsular polysaccharide synthesis enzyme Cap8G
YDGL002547	VF0003	Capsule cap8M	Capsular polysaccharide synthesis enzyme Cap8M
YDGL002015	VF0003	Capsule cap8O	Capsular polysaccharide synthesis enzyme Cap8O
YDGL002539	VF0003	Capsule cap8O	Capsular polysaccharide synthesis enzyme Cap8O
YDGL002014	VF0144	Capsule cps4I	UDP-*N*-acetylglucosamine-2-epimerase
YDGL002540	VF0144	Capsule cps4I	UDP-*N*-acetylglucosamine-2-epimerase
YDGL001235	VF0274	Capsule cpsJ	Glycosyl transferase CpsJ(V)
YDGL004140	VF0323	Capsule fcl	Putative fucose syntheses
YDGL002177	VF0323	Capsule glf	UDP-galactopyranose mutase
YDGL001165	VF0072	ClpC clpC	Endopeptidase Clp
YDGL002866	VF0072	ClpC clpC	Endopeptidase Clp
YDGL002842	VF0074	ClpP clpP	ATP-dependent Clp protease
YDGL001133	VF0215	Dispersin aatC	AatC ATB binding protein of ABC transporter
YDGL003303	VF0215	Dispersin aatC	AatC ATB binding protein of ABC transporter
YDGL003305	VF0215	Dispersin aatC	AatC ATB binding protein of ABC transporter
YDGL003564	VF0215	Dispersin aatC	AatC ATB binding protein of ABC transporter
YDGL003567	VF0215	Dispersin aatC	AatC ATB binding protein of ABC transporter
YDGL004218	VF0215	Dispersin aatC	AatC ATB binding protein of ABC transporter
YDGL002299	VF0273	Flagella fleQ	Transcriptional regulator FleQ
YDGL002756	VF0334	HSI-I PA0073	ATP-binding component of ABC transporter
YDGL002122	VF0159	Hsp60 htpB	Hsp60, 60K heat shock protein HtpB
YDGL004141	VF0367	LPS gmd	GDP-mannose 4,6-dehydratase
YDGL001251	VF0106	MgtBC mgtB	Mg^2+^transport protein
YDGL002018	VF0153	Mip mip	Macrophage infectivity potentiator (Mip)
YDGL003334	VF0298	MprAB mprA	MprB, sensor kinas. MprA, transcriptional factor
YDGL003892	VF0392	*O*-Antigen ddhA	Glucose-1-phosphate cytidylyltransferase
YDGL003891	VF0392	*O*-Antigen ddhB	CDP-glucose 4,6-dehydratase
YDGL003902	VF0392	*O*-Antigen galE	UDP-glucose 4-epimerase
YDGL003150	VF0319	PanC/PanD panD	panD
YDGL003842	VF0169	SodB sodB	Superoxide dismutase
YDGL003057	VF0101	Vi antigen tviB	Vi polysaccharide biosynthesis protein, UDP-glucose/GDP-mannose dehydrogenase

**Table 4:** Putative antibiotic resistance genes (A) identified in the genome of *Bacteroides fragilis* ZY-312, and minimum inhibitory concentration values (B) for each of the corresponding antibiotics.

Gene ID	Name	Drug
**(A)**		
YDGL002306	BcrA	Bacitracin
YDGL000449	BL2e_cepa	Cephalosporin
YDGL000258	dfrA22	Trimethoprim
YDGL003477	MefA	Macrolide
YDGL001150	MexF	Chloramphenicol, Fluoroquinolone
YDGL000604	MexY	Aminoglycoside, Glycylcycline
YDGL003440	RosA	Fosmidomycin
YDGL003171	tet37	Tetracycline
YDGL003704	VanRA	Vancomycin, Teicoplanin
YDGL003646	VatB	Streptogramin_A
YDGL000090	YkkC	na_antimicrobials

**Antibiotic**	**ZY-312**	**ATCC 25285**

**(B)**		
Cefoxitin	MIC = 2.0	MIC = 1.5
Ceftriaxone	MIC < 64	MIC < 64
Cefepime	MIC > 32	MIC > 32
Trimethoprim	MIC < 16	MIC < 16
Clarithromycin	MIC < 8	MIC < 8
Chloromycetin	MIC < 32	MIC < 32
Levofloxacin	MIC < 8	MIC < 8
Streptomycin	MIC > 1200	MIC > 1200
Kanamycin	MIC > 50	MIC > 50
Tetracycline	MIC < 16	MIC < 16
Vancomycin	4 < MIC < 8	4 < MIC < 8
Polymyxin B	MIC > 8	MIC > 8

*Bacitracin was eliminated because of high toxicity. Fosmidomycin is still being researched and was therefore excluded. Quinupristin/dalfopristin are not available in China and were also excluded. No corresponding antibiotic exists for the ykkC gene product.*

### Putative Virulence Factors

Through BLAST analysis of the VFDB, a total of 33 virulence factor homologs were identified in the genome of ZY-312 (**Table 3**). Most of these putative virulence genes encoded proteins involved in cellular structure or physiological activities, and none had previously been reported as being related to the pathogenesis of *B. fragilis*. Notably, *bft* was not present in the genome of ZY-312, indicating that it is a non-toxigenic strain.

### Antibiotic Resistance

Antibiotic resistance tests were performed based on the antibiotic resistance genes identified in the genome of YZ-312 (**Table 4A**), and results are summarized in **Table 4B**. Based on guidelines for antibiotic resistance breakpoints (Russell and Sambrook, 2002; National Center for Clinical Laboratories, 2006), ZY-312 showed resistance to cefepime, kanamycin, and streptomycin, but was susceptible to ceftriaxone, trimethoprim, clarithromycin, chloramphenicol, tetracycline, and levofloxacin. Without available guidelines for *B. fragilis*, resistance of ZY-312 to vancomycin and polymyxin B could not be confirmed; however, we observed that 4–8 μg/mL vancomycin and >8 μg/mL polymyxin B were sufficient to inhibit ZY-312 growth *in vitro*.

### ZY-312 Is Non-pathogenic in Both Normal and Immune-Deficient Mice

**FIGURE 1:**
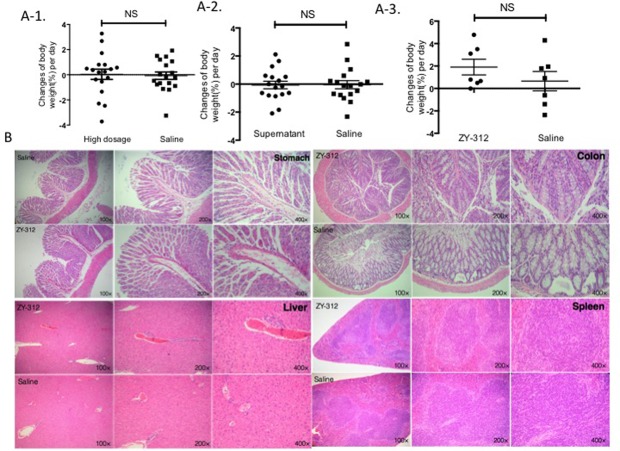
**ZY-312 is non-pathogenic in mice. (A)** Changes in body weight (%) per day were observed for the experimental and control groups. **(A-1)** Specific pathogen-free (SPF) BALB/c normal mice (*n* = 8) were treated with 5 × 10^11^ colony forming units (cfu)/day ZY-312 for 5 days and observed for 18 days. A control group was treated with saline. **(A-2)** SPF normal mice (*n* = 16) were treated with culture supernatant (0.5 mL/day) for 5 days and observed for 17 days. Tryptic soy broth was used for the control group. **(A-3)** Nude mice (*n* = 5) were treated with ZY-312 at a concentration of 1 × 10^9^ cfu/day for 3 days and observed for 7 days. The control group were treated with saline. **(B)** Light micrograph images of the stomach, colon, liver, and spleen from mice belonging to the high dosage group (upper) and control group (lower). No significant lesions were observed (mean ± SE; NS, not significant, *t*-test).

**FIGURE 2:**
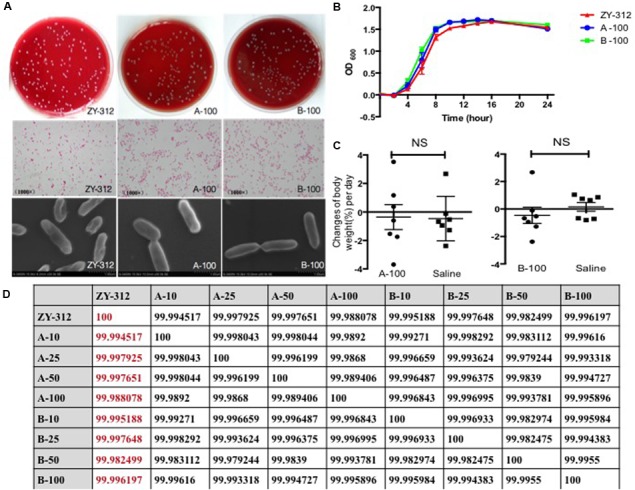
**ZY-312 is genetically stable. (A)** Gram-stained ZY-312, A_100_, and B_100_ cells following anaerobic culture on tryptic soy agar (5% sheep blood) for 48 h at 37°C. Observation were made using a light microscope (4000×) and a scanning electron microscope (30000×). **(B)** Growth curves of ZY-312, A_100_, and B_100_ cultured anaerobically for 24 h. **(C)** SPF BALB/c normal mice (*n* = 8) were treated with A_100_ or B_100_ at a concentration of 1 × 10^9^ cfu/day for 3 days and observed for 7 days. A control group was treated with saline. **(D)** Average nucleotide identities were calculated between each generation.

To confirm the *in vivo* safety of ZY-312, acute toxicity experiments were performed in both normal SPF BALB/c mice and nude mice. Lacking a thymus and an immune response, nude mice are an ideal animal model for evaluating probiotic safety. No death was observed in the BALB/c mice during the toxicity experiments, no treatment-related toxicity was observed, and no significant difference in body weight was noted between low, medium (**Supplementary Figure S4**), and high (**Figure 1A-1**) dose treatment groups and the control group, respectively. Similarly, no difference was found between the culture supernatant-treated group and the control group (**Figure 1A-2**). In addition, there was no obvious histopathological damage in the stomach, colon, liver, or spleen of BALB/c mice from the high dosage group (**Figure 1B**). There were also no significant differences in blood routine index or hepatorenal function between the high dose and control groups (data not shown). For the immune-deficient mouse toxicity experiments, ZY-312 again had no deleterious effects on body weight (**Figure 1A-3**).

### ZY-312 Is Genetically and Phenotypically Stable

To explore whether ZY-312 undergoes major genomic rearrangements during passage, the stability of ZY-312 was examined after 100 generations *in vitro*. A_100_ and B_100_ were morphologically identical to the original ZY-312 strain (**Figure 2A**) based on visual inspection, optical microscopy, and SEM. The growth characteristics of A_100_ and B_100_ (**Figure 2B**) were not significantly different from those of ZY-312. Furthermore, following oral administration of A_100_ or B_100_ at a dose of 1 × 10^9^ cfu/day for 3 days, mice did not show any clinical symptoms or weight loss during the observation period (**Figure 2C**). The calculated ANI values from each generation were at least 99.98% (**Figure 2D**).

## Discussion

According the recent reports, non-toxic *B. fragilis* now is regarded as a commensal constituent with potential as a probiotic candidate (Hsiao et al., 2013), because of powerful immunoregulatory benefits and health-promoting effects it owns. Although *B. fragilis* makes up a very small part of the human intestinal flora, it plays a unique role in the maturation of the host immune system (Mazmanian et al., 2005; Troy and Kasper, 2010). Mono-colonization of mice with *B. fragilis* was sufficient to correct systemic immune defects in germ-free mice by stimulating maturation of splenic CD4^+^ T cells (Mazmanian et al., 2005) and rebalancing the Th1/Th2 response. Furthermore, *B. fragilis* can direct an anti-inflammatory response, conferring protection in experimental mouse models of colitis (Mazmanian et al., 2008; Round and Mazmanian, 2010) and of autoimmune encephalomyelitis (Ochoa-Reparaz et al., 2010). Those observed effects were absent in mice treated with a *B. fragilis* PSA-deficient mutant strain. PSA is a dominant member of capsular polysaccharide complex (CPC) at the surface of *B. fragilis*, and contains a zwitterionic motif. *B. fragilis* is associated with clinical anaerobic infection and the CPC, especially PSA plays a key role in arising those diseases (Lindberg et al., 1982; Mazmanian and Kasper, 2006). Nevertheless, the role of PSA plays in intra-abdominal abscess probably is beneficial rather than harmful, since the inflammation aroused by PSA helps limit the spread of other gut bacteria and prevent more serious infection (Mazmanian and Kasper, 2006; Deng et al., 2016). Instead of being recognized as virulence factor, recently, PSA has been reconsidered as a symbiosis factor with health-promoting effect (Mazmanian et al., 2008).

Therefore, under overall considerations about the pros and cons, we believe non-toxic *B. fragilis* is a good choice for probiotic candidate and we decided to isolate a new strain of *B. fragilis* named ZY-312 (Deng et al., 2016) and explore its safety whether satisfy the criteria required for probiotic bacteria. Although the health benefits of *B. fragilis* are generally recognized, much work still needs to be done to obtain certification of this species as a probiotic. Probiotics are defined as non-pathogenic live microorganisms that confer health benefits to the host when administered in adequate amounts (FAO/WHO, 2002). As living microorganisms with the potential for infection or *in situ* toxin production, probiotics should fulfill health and safety claims before entering the market. Safety assessment is the chief task for certifying a probiotic, as any adverse effects should be predicted in advance. Accordingly (FAO/WHO, 2002; Miquel et al., 2015), correct identification, sufficient characterization, and evaluation of potential risk and probiotic properties are integral for evaluation of a new probiotic.

Previously, we demonstrated that novel strain ZY-312, isolated from the feces of a healthy breast-fed infant, possessed similar morphological and growth characteristics to typical *B. fragilis* strains, as well as exhibiting desirable probiotic properties, including tolerance to air, simulated gastric fluid (pH 3.0), simulated intestinal fluid and ox bile (pH 6.8), adhesion, and *in vitro* safety in colon cells (Deng et al., 2016). Based on these results, we carried out a more thorough characterization and systemic evaluation of ZY-312 in the current study. As recommended (FAO/WHO, 2002), phenotypic testing, fatty acid analysis, metabolite production, biochemical activity, and *in vivo* toxicity testing, in combination with genetic analysis, taxonomic identification, and putative virulence and antibiotic resistance gene identification analysis, were performed to determine whether ZY-312 is safe for use as a probiotic. The major fatty acids and metabolic products of ZY-312 closely resemble descriptions of *B. fragilis* in Bergey’s manual, as do the results of biochemical activity testing. Our previous work showed that the 16S rRNA sequence of ZY-312 was 99% identical to that of *B. fragilis* strain ATCC 28285. However, 16S rRNA sequence analysis has potential drawbacks for separating closely related species because of low taxonomic resolution. Whole genome sequencing identifies taxa with higher taxonomic resolution, and provides more information about gene function, such as putative virulence factors and antibiotic resistance genes (Rijkers et al., 2011; Miquel et al., 2015). Taking advantage of whole genome sequencing technology in the current study, we showed that ZY-312 and ATCC 25285 shared 99.99% ANI, and were derived from the same origin (**Supplementary Figure S5**), consistent with previous results. Furthermore, a total of 33 putative virulence factors and 11 antibiotic resistance genes were annotated in the genome of ZY-312 through comparative analysis with the VFDB and the ARDB, respectively. The putative virulence factors consisted of structural proteins and proteins with physiological activity, and none had previously been reported in association with the pathogenesis of *B. fragilis*. Because of the absence of *bft*, ZY-312 was identified as a NTBF strain.

There are many reports claiming probiotic safety based on an assessed lack of infectivity in normal animals (Bernardeau et al., 2002; Shokryazdan et al., 2016). However, confidence would be increased if assessment could be performed in immunodeficient animals (FAO/WHO, 2002). Therefore, we examined the safety of ZY-312 in both normal and immunodeficient mice. The SPF normal mice treated with high doses or supernatant of ZY-312 did not display any significant strain-related toxigenic symptoms, based on the assessment of body weight changes, histopathological examination, blood routine index, and hepatorenal function. Notably, ZY-312 also proved safe in nude mice (**Figure 1A-3**).

The FAO/WHO recommends that probiotic strains should be fully characterized, including determination of antibiotic resistance patterns, and should have no risk of transferring antibiotic resistance (FAO/WHO, 2002). Theoretically, consumption of probiotics with transferrable antibiotic resistance genes might lead to refractory infections if multiple antibiotic resistance genes are transferred to a pathogen (Borchers et al., 2009; Sanders et al., 2010). We demonstrated that there is no risk of ZY-312 spreading antibiotic resistance because all identified drug-resistance genes (**Table 4A**) were located in the chromosome rather than on a plasmid. Moreover, we verified the antibiotic resistance phenotype of ZY-312, and discovered that it is resistant to cefepime, kanamycin, and streptomycin, but susceptible to ceftriaxone, trimethoprim, clarithromycin, chloramphenicol, tetracycline, and levofloxacin. The MICs of vancomycin and polymyxin B for ZY-312 were also identified (**Table 4B**). The inconsistencies between genotype and observed phenotype might stem from the fact that putative antibiotic resistance genes were annotated based on 40% amino acid homology, meaning that some genes were incorrectly identified in the genome of ZY-312, and are likely not present.

As potential genetic variation might lead to unpredictable risk, genetic stability should also be confirmed prior to starting large-scale production of ZY-312-based probiotics (Sanders et al., 2010). We confirmed that following *in vitro* passage of 100 generations from the original strain of ZY-312, there was no significant difference between A_100_, B_100_, and the parental strain with respect to morphological characteristics and growth features. No *in vivo* toxicity was observed for A_100_ or B_100_, and the ANI between each generation was at least 99.98%, demonstrating that ZY-312 has a high degree of genetic stability.

## Conclusion

We confirmed that ZY-312 is a NTBF strain, without potential virulence factors or risk of spreading antibiotic resistance genes. As well as having desirable probiotic properties (Deng et al., 2016), ZY-312 has a high degree of genetic stability and is non-pathogenic, even to immune-deficient mice. Therefore, ZY-312 is most likely safe for use in future probiotic applications. This study supplements the initial safety assessment work already carried out for ZY-312, and contributes to the development of the first probiotic representative from the dominant Bacteroidetes phylum.

## Author Contributions

YW did the experiments with DNA and bacteria, analyzed data, and contributed to revising the manuscript; HD did the experiments with bacteria and mice, analyzed data, and wrote the manuscript; ZL did the experiments with mice, analyzed data, and contributed to revising the manuscript; YT, YH, XW, and ZD analyzed data; YL and RY designed the experiments and contributed to revising the manuscript; YjB designed experiments, analyzed data, and provided overall direction, YB and FZ provided overall directions and contributed to revising the manuscript.

## Conflict of Interest Statement

The authors declare that the research was conducted in the absence of any commercial or financial relationships that could be construed as a potential conflict of interest. The property of ZY-312 belongs to Guangzhou ZhiYi biotechnology Co. Ltd. Any use of ZY-312 without permission of Guangzhou ZhiYi biotechnology Co. Ltd. will be illegal.

## Footnotes

1 https://github.com/jstjohn/SeqPrep

2 https://github.com/najoshi/sickle

3 http://ardb.cbcb.umd.edu/

4 http://www.mgc.ac.cn/VFs/main.htm

## Supplementary Material

FIGURE S1 **(A)** Colonies of ZY-312 on tryptone soy agar (5% sheep blood) following culture in 5% CO_2_ **(A-A)**, air **(A-B)**, or anaerobically **(A-C)** for 48 h at 37°C. **(B)** Cells observed under light microscope following Gram staining (4000×). **(C)** Cells observed under scanning electron microscope (5000×).

FIGURE S2 **Confirmation of no microbial contamination. (A)** *Escherichia coli* and ZY-312 cultured on Eosin methylene blue agar for 24 h at 37°C. **(B)** *Salmonella enterica* serovar Paratyphi B and ZY-312 cultured on SS agar for 24 h at 37°C. **(C)** *Clostridium sporogenes* and ZY-312 cultured anaerobically on Columbia agar containing blood and gentamicin for 48 h at 37°C. **(D)** *Staphylococcus aureus* and ZY-312 cultured on mannitol sodium chloride agar for 24 h at 37°C. **(E)** *Pseudomonas aeruginosa* and ZY-312 cultured on NAC agar for 24 h at 37°C. **(F)** *Candida albicans* and ZY-312 cultured on Sabouraud dextrose agar for 72 h at 28°C. **(G)** *S. aureus* and ZY-312 cultured on agar agar for 48 h at 37°C. **(H)** *C. albicans* and ZY-312 cultured on rose bengal agar for 96 h at 28°C.

FIGURE S3 **The major fatty acids of ZY-312 as identified by gas chromatography**.

FIGURE S4 **Acute toxicity of ZY-312 to mice. Changes in body weight (%) per day were observed for the experimental and control groups. (A)** Specific pathogen-free (SPF) BALB/c normal mice (*n* = 5) were treated with ZY-312 at a concentration of 1 × 10^9^ cfu/day for 5 days and observed for 15 days. The control group mice were treated with saline. **(B)** SPF normal mice (*n* = 5) were treated with ZY-312 at a concentration of 5 × 10^10^ cfu/day for 5 days and observed for 18 days. Tryptic soy broth was used to treat the control group. No significant weight loss was observed in any of the animals (mean ± SE; NS, not significant, *t*-test).

FIGURE S5 **Phylogenetic tree based on complete shotgun sequences showing the relationship between *Bacteroides fragilis* ZY-312 and closely related species.** The tree was constructed using the neighbor-joining method.

FIGURE S6 **Phylogenetic tree based on complete genome sequences showing the relationship between *B. fragilis* strain ZY-312, *B. fragilis* NCTC 9343, *B. fragilis* YCH46, and *B. fragilis* 638R.** The tree was constructed using the neighbor-joining method.
