# Supplementary figures and images for: Safety Evaluation of a Novel Strain of Bacteroides fragilis
Source: Front Microbiol. 2017 Mar 17;8:435. doi: 10.3389/fmicb.2017.00435 (PMC5355466; doi:10.3389/fmicb.2017.00435)

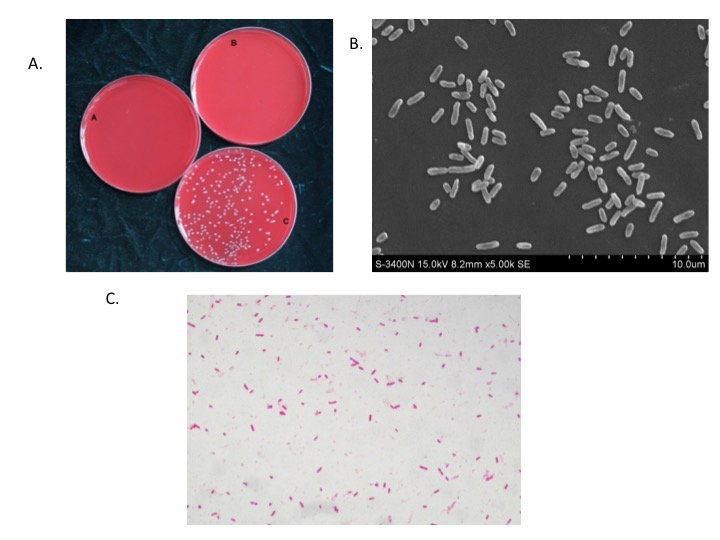

Supplement: FIGURE S1 — (A) Colonies of ZY-312 on tryptone soy agar (5% sheep blood) following culture in 5% CO2 (A-A), air (A-B), or anaerobically (A-C) for 48 h at 37°C. (B) Cells observed under light microscope following Gram staining (4000×). (C) Cells observed under scanning electron microscope (5000×). [file Image_1.JPEG]

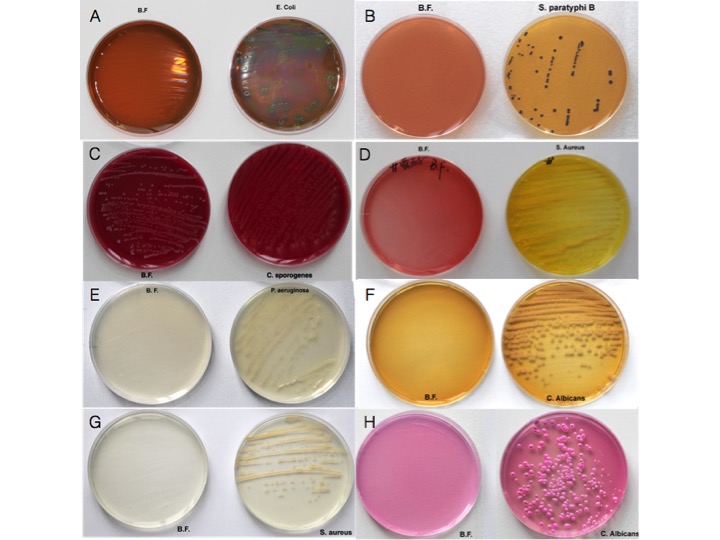

Supplement: FIGURE S2 — Confirmation of no microbial contamination. (A) Escherichia coli and ZY-312 cultured on Eosin methylene blue agar for 24 h at 37°C. (B) Salmonella enterica serovar Paratyphi B and ZY-312 cultured on SS agar for 24 h at 37°C. (C) Clostridium sporogenes and ZY-312 cultured anaerobically on Columbia agar containing blood and gentamicin for 48 h at 37°C. (D) Staphylococcus aureus and ZY-312 cultured on mannitol sodium chloride agar for 24 h at 37°C. (E) Pseudomonas aeruginosa and ZY-312 cultured on NAC agar for 24 h at 37°C. (F) Candida albicans and ZY-312 cultured on Sabouraud dextrose agar for 72 h at 28°C. (G) S. aureus and ZY-312 cultured on agar agar for 48 h at 37°C. (H) C. albicans and ZY-312 cultured on rose bengal agar for 96 h at 28°C. [file Image_2.JPEG]

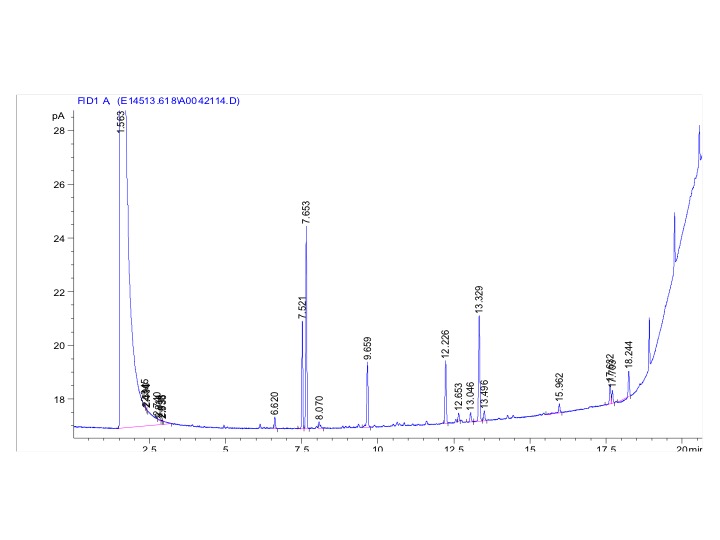

Supplement: FIGURE S3 — The major fatty acids of ZY-312 as identified by gas chromatography. [file Image_3.JPEG]

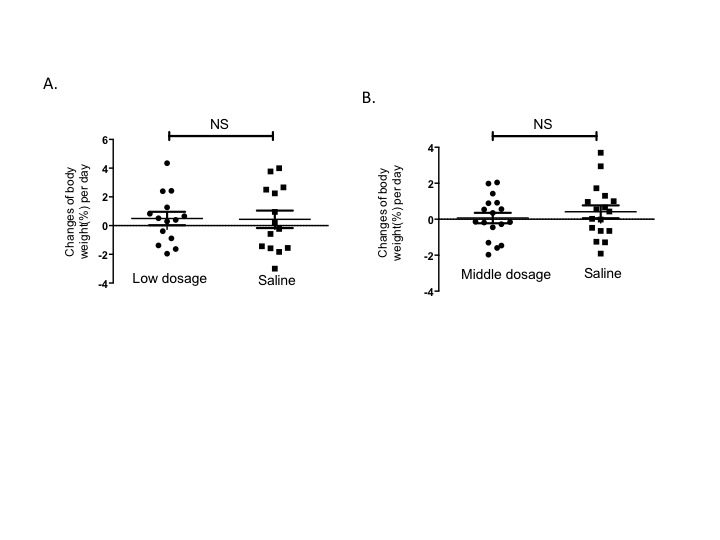

Supplement: FIGURE S4 — Acute toxicity of ZY-312 to mice. Changes in body weight (%) per day were observed for the experimental and control groups. (A) Specific pathogen-free (SPF) BALB/c normal mice (n = 5) were treated with ZY-312 at a concentration of 1 × 10^9^ cfu/day for 5 days and observed for 15 days. The control group mice were treated with saline. (B) SPF normal mice (n = 5) were treated with ZY-312 at a concentration of 5 × 10^10^ cfu/day for 5 days and observed for 18 days. Tryptic soy broth was used to treat the control group. No significant weight loss was observed in any of the animals (mean ± SE; NS, not significant, t-test). [file Image_4.JPEG]

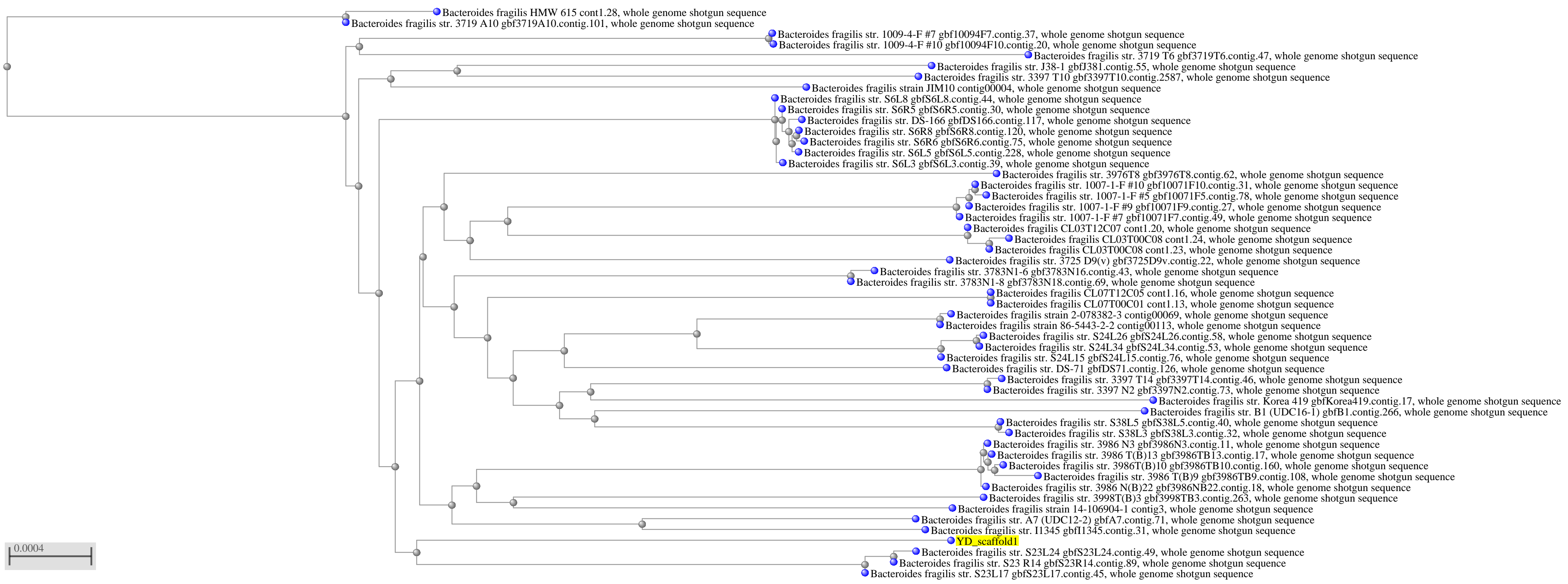

Supplement: FIGURE S5 — Phylogenetic tree based on complete shotgun sequences showing the relationship between Bacteroides fragilis ZY-312 and closely related species. The tree was constructed using the neighbor-joining method. [file Image_5.PDF]

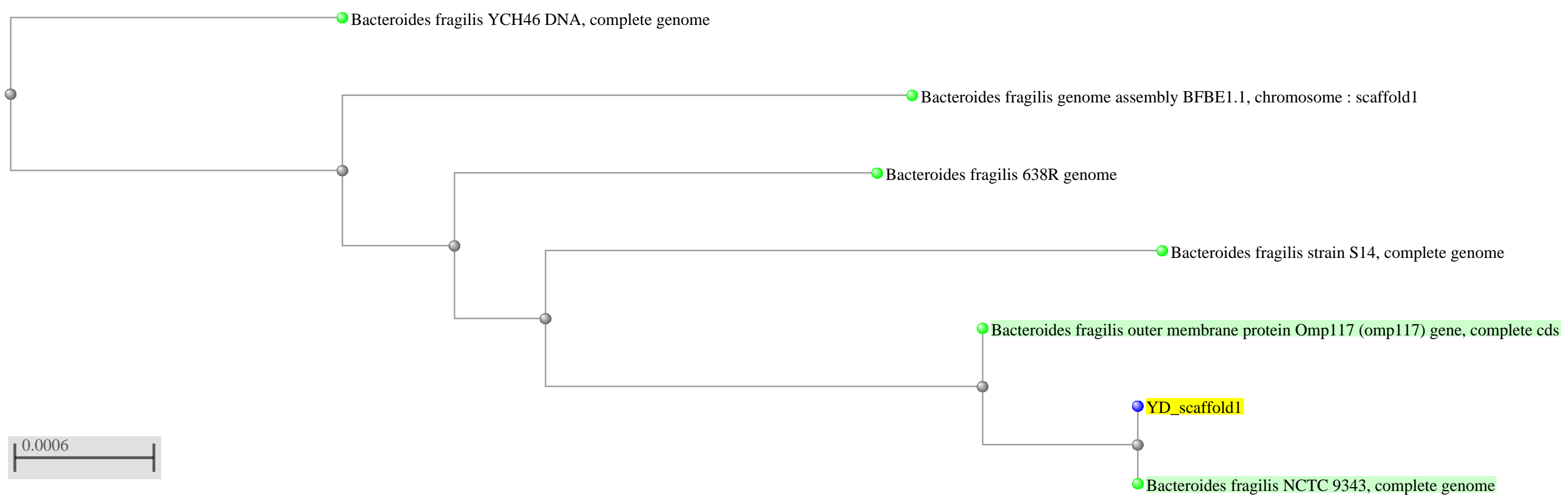

Supplement: FIGURE S6 — Phylogenetic tree based on complete genome sequences showing the relationship between B. fragilis strain ZY-312, B. fragilis NCTC 9343, B. fragilis YCH46, and B. fragilis 638R. The tree was constructed using the neighbor-joining method. [file Image_6.PDF]
